# An Efficient Procedure to Compensate for the Errors Due to the Probe Mispositioning in a Cylindrical Near-Field Facility

**DOI:** 10.3390/s24061787

**Published:** 2024-03-10

**Authors:** Florindo Bevilacqua, Francesco D’Agostino, Flaminio Ferrara, Claudio Gennarelli, Rocco Guerriero, Massimo Migliozzi, Giovanni Riccio

**Affiliations:** 1 Dipartimento di Ingegneria Industriale, Università di Salerno, Via Giovanni Paolo II, I-84084 Fisciano, Italy; fbevilacqua@unisa.it (F.B.); fdagostino@unisa.it (F.D.); flferrara@unisa.it (F.F.); rguerriero@unisa.it (R.G.); mmigliozzi@unisa.it (M.M.); 2 Dipartimento di Ingegneria dell’Informazione ed Elettrica e Matematica Applicata, Università di Salerno, Via Giovanni Paolo II, I-84084 Fisciano, Italy; griccio@unisa.it

**Keywords:** antennas characterization, near-to-far-field transformation techniques, non-redundant sampling representation, cylindrical scan, probe mispositioning errors correction

## Abstract

This paper deals with the compensation of the probe mispositioning errors occurring in a cylindrical near-field (NF) facility due to the imprecise control of the linear and azimuthal positioners allowing the cylindrical scanning and/or to their limited resolution and to defects in the rails guiding the linear motion. As a result, 3-D errors in the positioning of the probe at any sampling point, as prescribed by the adopted non-redundant representation, affect the accuracy of the NF measurements. An efficient procedure is here proposed to properly compensate for these errors. It involves two steps. The former allows one to correct the mispositioning errors due to the deviation of each actual sampling point from the nominal measurement cylinder. The latter makes use of an iterative technique to restore the NF samples at any sampling point fixed by the used non-redundant representation from the ones obtained at the previous step and affected by 2-D mispositioning errors. Once these steps have been fruitfully applied, the so-compensated NF samples are effectively interpolated through a 2-D optimal sampling interpolation (OSI) formula to accurately reconstruct the input data required to perform the traditional cylindrical near-to-far-field transformation. The OSI representation is here developed by considering an elongated antenna under test as enclosed either in a prolate spheroid or in a cylinder terminated by two half spheres (rounded cylinder) in order to make the representation effectively non-redundant. Numerical test results, which thoroughly prove the efficacy of the devised procedure in correcting even severe 3-D mispositioning errors, are reported.

## 1. Introduction

As known, near-to-far-field (NTFF) transformation techniques play a key role in modern antenna measurements [1,2,3,4,5,6,7,8,9,10,11]. The characteristics of the antenna under test (AUT) (frequency, electric size, far-field angular coverage, etc.) and the intended application determine the choice of the NTFF transformation technique to be used, which guarantees the greatest accuracy [1,11]. Among them, the NTFF transformations using the cylindrical scanning geometry [12,13,14,15,16,17,18,19,20,21,22,23] are particularly appealing when characterizing an AUT that mainly radiates in an angular region centred on the horizontal plane. For instance, this occurs in the characterization of the antennas used for radio base stations [24,25] or for body-centric communications [26]. These transformations make use of NF data collected at given sampling points on a cylindrical surface, whose locations are reached by matching the azimuthal and linear movements of the AUT and measuring probe, respectively. In the conventional approach [12,13,14], the number of sampling points is related to the height of the scanning cylinder, the maximum transverse dimension of the AUT, the spectral content of the AUT field in its radiating NF region, and the employed fast Fourier transform (FFT) algorithm exploited to efficiently evaluate the modal coefficients of the cylindrical wave expansion required to reconstruct the FF pattern.

In this context, effective cylindrical NTFF transformation techniques, which make use of a reduced number of NF measurements, were devised in [17,18] by a suitable application of the key results concerning the non-redundant (NR) representations of the electromagnetic (EM) fields [27] to the voltage detected at the measuring probe terminals. These techniques allow one to remarkably save measurement time as compared to the conventional one [12,13,14]. In fact, while in the conventional approach, the distance between the sampling rings is *λ* or smaller (*λ* being the wavelength at the working frequency), in the NR sampling ones, it increases moving from the central to the peripheral zones. Moreover, unlike the conventional approach, which requires acquiring on each ring the same amount of NF data related to the radius of the smallest cylinder containing the AUT, this number decreases as it moves from the central rings to the peripheral ones. The radiation pattern of the AUT is then accurately calculated through the conventional transformation [12,14], whose massive input NF data are attained by interpolating the gathered NR samples by means of optimal sampling interpolation (OSI) formulae. The sample arrangement depends on the surface chosen to model the AUT, besides the radius and height of the scanning cylinder. The geometry of the antennas usually characterized in a cylindrical NF facility is of elongated type, and, to fit it as much as possible, two efficient modeling surfaces can be adopted [17,18]: the surface bounding a cylinder terminated by two half spheres (rounded cylinder) and that bounding a prolate spheroid. As a useful choice criterion, prolate spheroidal modeling can be conveniently chosen when the transverse section of the AUT has its maximum in its central region; otherwise, the rounded cylinder reduces the volumetric redundancy.

The accuracy of the FF reconstruction depends on the precision of the NF measurements. A source of inaccuracy is the mispositioning of the probe at each prescribed sampling point. In a cylindrical NF facility, the azimuthal movement of the AUT occurs through a rotating table, while the linear motion of the probe is allowed by a vertical positioner. The imprecise control of these positioners, as well as their limited resolution, can impair the accuracy of the NF measurements since the sampling point’s position on the cylindrical surface, as prescribed by the adopted sampling representation, can differ from that actually reached by these positioners. Furthermore, imperfections in the mechanical rails guiding the linear motion of the probe may cause deviations from the radius preset for the scanning. As a consequence, the NF measurements are affected by mispositioning errors that, due to their characteristics, are three-dimensional (3-D). Fortunately, the availability of laser interferometric techniques makes it possible to determine the actual reached positions and estimate the amount of positioning errors. A similar issue occurs when considering in situ characterizations using unmanned aerial vehicles [28,29], due to uncertainties in the inertial navigation system and the weather conditions affecting the flight trajectory [30]. In such a case, the sampling locations can be determined by means of the GPS. This kind of error has been corrected in the case of classical NTFF transformations with plane-rectangular, spherical, and cylindrical scannings by adopting a matrix formulation [31,32,33]. However, such an approach is not suitable for the here-considered NR cylindrical NTFF transformation.

In such a case, the 3-D mispositioning errors can be effectively compensated for by devising a strategy that resembles the approach derived and profitably applied to correct such errors affecting the NR NTFF transformations with planar scans [34,35]. It involves two steps. The former consists of a phase correction to compensate for the probe mispositioning errors arising from the deviations of the acquired NF samples from the preset scanning cylinder. It is denoted as cylindrical wave correction in the following and is quite similar to that proposed in [36] to compensate for the plane distance deviations in the planar scans. The NR data, restored by applying the previous step, were corrupted only by 2-D position errors. Then, the exactly positioned (uniform) samples can be retrieved from these by resorting to a simple and flexible procedure based on an iterative technique successfully applied to cylindrical [37], spherical [38], and non-conventional plane-rectangular [39] scannings. It is noteworthy that such a procedure converges only if there exists a one-to-one correspondence linking the uniform sampling points to the corresponding incorrectly positioned (non-uniform) closest ones. The adopted iterative-based procedure is preferable to the alternative one in [37,38,39], which exploits the singular value decomposition (SVD) method to recover the uniform samples from those affected by positioning errors and does not suffer from the above drawback. In fact, in order for the SVD-based method to be successfully applied, it is required that the 2-D problem of the uniform sample reconstruction be reduced to two independent 1-D problems, thus avoiding a considerable computational effort. However, the here-considered error compensation problem does not always allow one to fulfill such a constraint. Any interested reader can refer to [38] for a complete bibliography and a comprehensive discussion on the reconstruction of the uniform samples from those non-uniformly positioned.

The goal of this article is to develop NR NTFF transformations with cylindrical scans from NF samples corrupted by 3-D probe mispositioning errors and to prove their numerical feasibility. For this purpose, the two-step procedure is conveniently applied to compensate for such errors when the NF sampling arrangement is determined by adopting both the prolate spheroid and the rounded cylinder modeling. In order to highlight the novelty and usefulness of the proposed technique, it is perhaps worthy to stress that the matrix formulation in [31,32,33] cannot be applied in the NR NTFF transformations framework, as well as the approaches in [37,38,39], since they require that the NF samples lie on the scanning surface.

The article is organized according to the following sections. Section 2 describes how to develop the effective sampling representation of the voltage measured by the probe on the scan cylinder by conveniently applying the non-redundant representations of EM fields [27] and considering an elongated AUT as contained either in a prolate spheroid or in a rounded cylinder. The same section also describes the 2-D OSI algorithm to be used for recovering the probe voltage at any point on the scan cylinder from a NR number of its samples. Section 3 presents the developed two-step procedure to compensate for the 3-D probe mispositioning errors. Section 4 reports many numerical results assessing the effectiveness of the devised compensation procedure. Finally, Section 5 collects concluding remarks.

## 2. OSI Representation over a Cylinder from a NR Number of NF Samples

The key results regarding the development of an effective probe voltage representation over a cylindrical surface from a reduced number of its uniformly spaced NF samples, particularly convenient when characterizing elongated antennas, and the related 2-D OSI formula are here summed up for the reader’s convenience.

**Figure 1:**
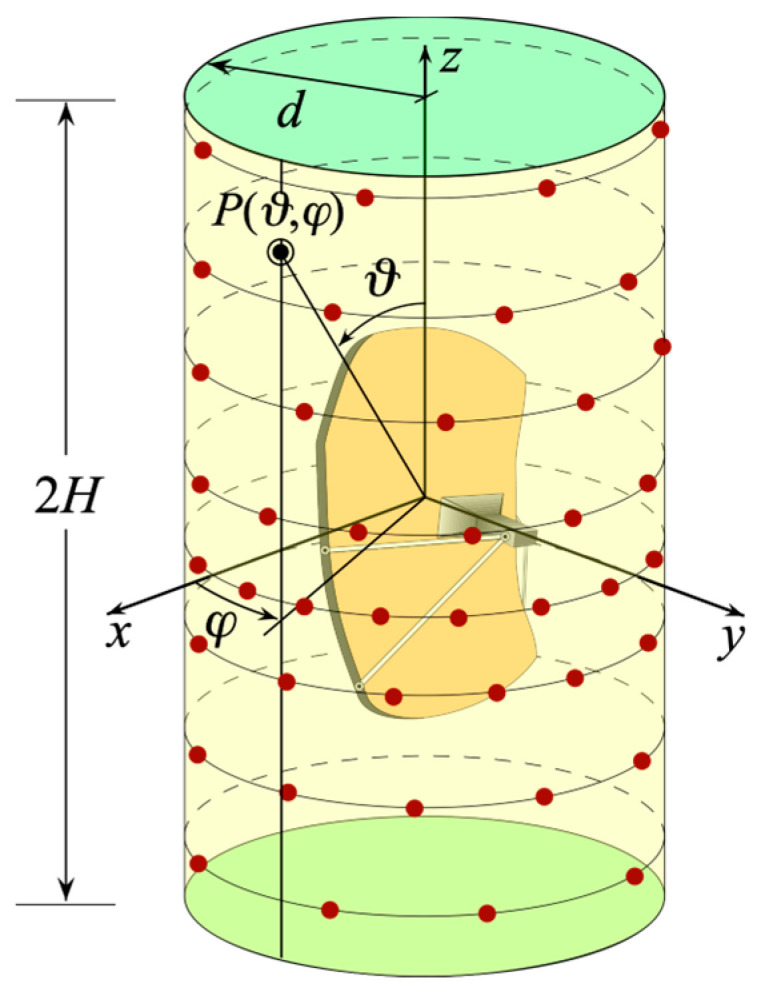
Cylindrical scanning for a long AUT.

To this end, let us introduce a cylindrical coordinate system (*ρ*, *φ*, *z*), with its *z*-axis coincident with the axis of a scanning cylinder of radius *d* and height 2*H* and origin *O* at the center of the considered elongated AUT, to denote any observation point *P* on the cylindrical surface (see Figure 1). Moreover, let us suppose, as usual in practice, that we adopt a non-directive probe to gather the required NF data. The probe characteristics enable us to apply the results relevant to the NR representations [27] to the gathered voltage too, since the voltage measured at its terminals has the same effective spatial bandwidth as the field [40]. According to these results, it is necessary to adopt a convenient convex surface $S_{M}$ to model the considered long AUT and find the optimal parameter *σ* to describe any of the azimuthal rings and generatrices representing the scan cylinder. Moreover, the expressions of the probe and rotated probe voltages $V=V_{p,r}(\sigma )$ must be multiplied by a proper phase factor $e^{j\gamma (\sigma )}$ in order to introduce the so named “reduced voltage”

(1) $$\tilde{V}(\sigma )=V(\sigma )e^{j\gamma (\sigma )}$$

which has been shown [27] to be a spatially quasi-bandlimited, but not strictly bandlimited, function. Thus, its approximation with a bandlimited function gives rise to an unavoidable error. Such an error can be minimized by making its bandwidth $BW_{\sigma }$ greater than a threshold [27]. Hence, an approximation function with an enlarged bandwidth $\chi^{\prime }BW_{\sigma }$ can be adopted to this end, where *χ*^′^ > 1 indicates the excess bandwidth factor.

According to [27], a NR representation of the voltage on a cylinder generatrix is achieved by choosing the bandwidth equal to

(2) $$BW_{\sigma }=\ell^{\prime }/\lambda$$

where $\ell^{\prime }$ is the length of the curve *C*^′^, got as intersection of the meridian plane through *P* with $S_{M}$, and adopting as optimal parameter and phase function:

(3) $$\sigma =\left(\pi /\ell^{\prime }\right)\left[\;\bar{PP_{1}}-\bar{PP_{2}}+\mu_{1}^{\prime }+\mu_{2}^{\prime }\right]$$

(4) $$\gamma =\left(\pi /\lambda \right)\left[\;\bar{PP_{1}}+\bar{PP_{2}}+\mu_{1}^{\prime }-\mu_{2}^{\prime }\right]$$

where $\mu_{1,2}^{\prime }$ are the arclength abscissae of the tangency points $P_{1,2}$ on *C*^′^ and $\bar{PP_{1}}$ and $\bar{PP_{2}}$ the distances from *P* to $P_{1,2}$.

For an azimuthal ring of radius *ρ*, the angle *φ* is conveniently used as parameter and *γ* can be considered constant and equal to that used for the generatrix through *P*. Moreover, the related bandwidth can be evaluated by [27]:

(5) $$BW_{\varphi }=\left(\pi /\lambda \right)\underset{z^{\prime }}{\max}(R^{+}-R^{-})=\left(\pi /\lambda \right)\underset{z^{\prime }}{\max}(\sqrt{{\left(\rho +\rho^{\prime }(z^{\prime })\right)}^{2}+{\left(z-z^{\prime }\right)}^{2}}-\sqrt{{\left(\rho -\rho^{\prime }(z^{\prime })\right)}^{2}+{\left(z-z^{\prime }\right)}^{2}})$$

where *ρ*’(*z*’) is the equation of $S_{M}$ in cylindrical coordinates and $R^{+}$ and $R^{-}$ are the maximum and minimum distances between the scanning ring and the circumference of radius *ρ*’ lying on $S_{M}$ by the same side of the ring.

Depending on the AUT geometry, $S_{M}$ can be chosen as the surface bounding a prolate spheroid or a rounded cylinder, in order to reduce as much as possible the volumetric redundancy.

**Figure 2:**
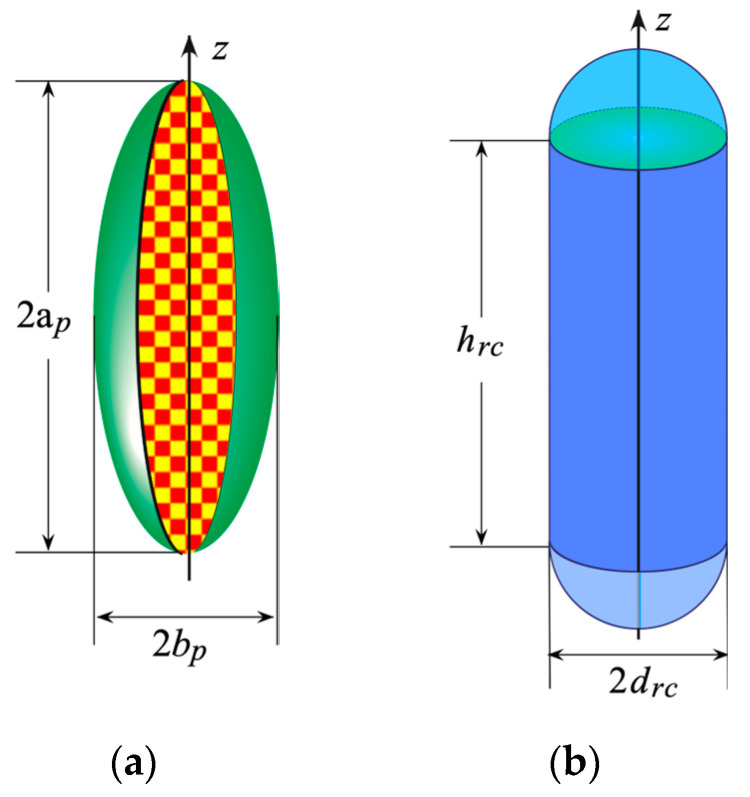
(**a**) Prolate spheroid. (**b**) Rounded cylinder.

When the transverse section of the long AUT has its maximum in its central region, then the best choice is to fit it by a prolate spheroid (Figure 2a), having its semi-major and semi-minor axes equal to $a_{p}$ and $b_{p}$ respectively. Otherwise, when the considered AUT has a regular geometry, a rounded cylinder (Figure 2b), made by a cylinder $h_{rc}$ high ending in two hemispheres having radius $d_{rc}$, allows one to reduce the volumetric redundancy.

In the former case [18], $\ell^{\prime }=4a_{p}E\left(\pi /2|\varepsilon^{2}\right)$ and relations from (3) to (5) become:

(6) $$\sigma =\frac{\pi }{2}\left[1+\frac{E\left(\sin^{-1}u_{p}|\varepsilon^{2}\right)}{E\left(\pi /2|\varepsilon^{2}\right)}\right];\;\gamma =\frac{2\pi a_{p}}{\lambda }\left[v\sqrt{\frac{{v_{p}}^{2}-1}{{v_{p}}^{2}-\varepsilon^{2}}}-E\left(\cos^{-1}\sqrt{\frac{1-\varepsilon^{2}}{{v_{p}}^{2}-\varepsilon^{2}}}|\varepsilon^{2}\right)\right]$$

(7) $$BW_{\varphi }=\frac{2\pi b_{p}}{\lambda }\sin\vartheta_{\infty }\left(\sigma \right)$$

where *E*(•|•) denotes the elliptic integral of second kind, $\varepsilon =f/a_{p}$ is the eccentricity of the intersection ellipse between a meridian plane and $S_{M}$, 2*f* its focal distance, $u_{p}=(r_{1}-r_{2})/2f$ and $v_{p}=(r_{1}+r_{2})/2a_{p}$ the related elliptic coordinates, $r_{1,2}$ being the distances from observation point *P* to the ellipse foci, and $\vartheta_{\infty }=\sin^{-1}(u_{p})+\pi /2$ is the polar angle of the asymptote to the hyperbola through any point on the ring.

**Figure 3:**
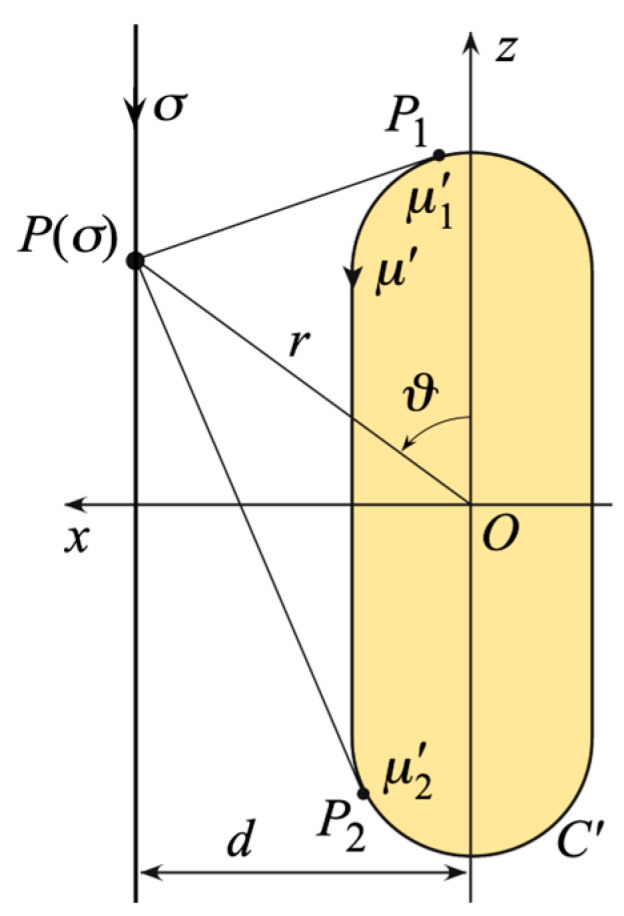
Relevant to the evaluation of *σ* and *γ* when using the rounded cylinder modeling.

In the latter case [17], $\ell^{\prime }=2(h_{rc}+\pi d_{rc})$, the parameters in (3) and (4) can be evaluated (see Figure 3) by means:

(8) $$\bar{PP_{1,2}}=\sqrt{{\left(z\mp h_{rc}/2\right)}^{2}+d^{2}-{d_{rc}}^{2}};\;\mu_{1}^{\prime }=d_{rc}\sin^{-1}\left(\frac{d_{rc}d+\bar{PP_{1}}\left((h_{rc}/2)-z\right)}{{\bar{PP_{1}}}^{2}+{d_{rc}}^{2}}\right)$$

(9) $$\mu_{2}^{\prime }=h_{rc}+d_{rc}\left[\pi -\sin^{-1}\left(\frac{d_{rc}d+\bar{PP_{2}}\left((h_{rc}/2)+z\right)}{{\bar{PP_{2}}}^{2}+{d_{rc}}^{2}}\right)\right]$$

and the maximum in (5) is attained at

(10) $$z^{\prime }=\{\begin{array}{cc} z & \mathrm{if}\left|z\right|\leq h_{rc}/2\\ \left[\frac{h_{rc}}{2}+\frac{\left(\left|z\right|-h_{rc}/2\right){d_{rc}}^{2}}{d^{2}+{\left(\left|z\right|-h_{rc}/2\right)}^{2}}\right]\mathrm{sgn}(z) & \mathrm{if}\left|z\right|>h_{rc}/2 \end{array}$$

where sgn (•) denotes the sign function.

Whatever modeling is used to develop the sampling representation and, therefore, the sampling arrangement over the cylinder, the next 2-D OSI expansion [17,18], attained by combining the 1-D ones along the rings and generatrices, is conveniently utilized to recover the voltage value *V* at any point *P* on the measurement surface, as well as at those of the standard cylindrical lattice:

(11) $$\tilde{V}(\sigma ,\varphi )=\sum_{n=n_{0}-q+1}^{n_{0}+q}\{SIF\left(\sigma ,\sigma_{n},\bar{\sigma },N,N^{\prime \prime }\right)\sum_{m=m_{0}-p+1}^{m_{0}+p}\tilde{V}\left(\sigma_{n},\varphi_{m,n}\right)SIF\left(\varphi ,\varphi_{m,n},{\bar{\varphi }}_{n},M_{n},M_{n}^{\prime \prime }\right)\}$$

where $n_{0}=\mathrm{floor}\left(\sigma /\Delta \sigma \right)$, $m_{0}=\;\mathrm{floor}\left(\varphi \;/\Delta \varphi_{n}\right)$, 2p, 2q are the numbers of reduced voltages samples $\tilde{V}\left(\sigma_{n},\varphi_{m,n}\right)$ considered in the interpolations along the involved rings and the generatrix at *φ*,

(12) $$\sigma_{n}=n\Delta \sigma =\frac{2\pi n}{2N^{\prime \prime }+1};\;\varphi_{m,n}=m\Delta \varphi_{n}=\frac{2m\pi }{2M_{n}^{\prime \prime }+1}$$

(13) $$\;N^{\prime \prime }=\mathrm{floor}\left(\chi N^{\prime }\right)+1;\;N^{\prime }=\mathrm{floor}\left(\chi^{\prime }W_{\sigma }\right)+1;\;N=N^{\prime \prime }-N^{\prime }$$

(14) $$M_{n}^{\prime \prime }=\mathrm{floor}\left(\chi M_{n}^{\prime }\right)+1;\;M_{n}^{\prime }=\mathrm{floor}\left(\chi^{*}W_{\varphi_{n}}\right)+1$$

(15) $$M_{n}=M_{n}^{\prime \prime }-M_{n}^{\prime };\;\bar{\sigma }=q\Delta \sigma ;\;{\bar{\varphi }}_{n}=p\Delta \varphi_{n}$$

$\chi^{*}=1+(\chi^{\prime }-1){\left[\sin\vartheta (\sigma_{n})\right]}^{-2/3}$ and *χ* > 1 are the azimuthal bandwidth excess and oversampling factors [27], and floor(•) is the floor function. Furthermore, in (11)

(16) $$SIF\left(x,x_{i},\bar{x},J,J^{\prime \prime }\right)=Tch_{J}\left[(x-x_{i}),\bar{x}\right]Dirch_{J^{\prime \prime }}\left(x-x_{i}\right)$$

is the OSI kernel, wherein

(17) $$Tch_{J}\left(x,\bar{x}\right)=\frac{T_{J}\left[2\cos^{2}\left(x/2\right)/\cos^{2}\left(\bar{x}/2\right)-1\right]}{T_{J}\left[2/\cos^{2}\left(\bar{x}/2\right)-1\right]};\;Dirch_{J^{\prime \prime }}\left(x\right)=\frac{\sin\left[(2J^{\prime \prime }+1)x/2\right]}{\left(2J^{\prime \prime }+1)\sin(x/2\right)}$$

represent the Tschebyscheff and the Dirichlet smart window functions, respectively, being $T_{J}(\cdot )$ the *J* degree Tschebyscheff polynomial.

## 3. Procedure to Correct 3-D Probe Mispositioning Errors

Let us assume that the gathered NF data are not regularly spaced on the measurement cylinder and that the positioning of each sampling point suffers from a radial deviation with respect to the radius of the prescribed surface. Therefore, the NF measurements are corrupted by 3-D probe mispositioning errors, whose amount is known if laser interferometric techniques are used to estimate them. In such a case, these 3-D errors can be effectively compensated for by adopting an effective strategy resembling that in [34,35] and consisting of two steps.

The former step consists of performing a phase correction to compensate for the mispositioning errors due to defects in the rail guiding the probe, which entail deviations of the actual radial coordinates of the sampling points from those defining the measurement cylinder. Due to the geometry of the problem, such a correction has been denoted as a cylindrical wave correction. Accordingly, if $\delta_{r}$ denotes, for r=1,…,R, the deviation of each of the *R*-gathered voltage samples from the surface of the scan cylinder, then the following relation

(18) $$V(d,\varphi ,z)=V(d+\delta_{r},\varphi ,z)e^{j2\pi \delta_{r}/\lambda }$$

can be used to reconstruct the voltage samples which would be acquired at the exact radial coordinate *d* from those, $V(d+\delta_{r},\varphi ,z)$, altered by the 3-D mispositioning errors. At this stage, the so obtained NF data result to be non-uniformly spaced over the preset scan surface and, therefore, corrupted only by 2-D position errors.

The latter step involves the reconstruction, from the previously determined NF data, of the NF samples at the points set by the sampling representation based either on the prolate spheroid or the rounded cylinder modeling. To this end, the procedure [37] based on the iterative technique can be used. This last requires to be successfully applied that there exists a one-to-one correspondence linking the uniform sampling points to the corresponding ‘‘closest’’ non-uniform ones. This requirement can be considered well satisfied by the problem being addressed. Hence, it is possible to express, through the 2-D OSI formula (11), the reduced voltages at the non-uniform sampling points $\left(\xi_{k},\alpha_{j,k}\right)$ in terms of the unknown samples at the closest uniform ones $\left(\sigma_{n},\varphi_{m,n}\right)$. Hence, it results:

(19) $$\tilde{V}(\xi_{k},\alpha_{j,k})=\sum_{n=n_{0}-q+1}^{n_{0}+q}\{SIF\left(\xi_{k},\sigma_{n},\bar{\sigma },N,N^{\prime \prime }\right)\sum_{m=m_{0}-p+1}^{m_{0}+p}\tilde{V}\left(\sigma_{n},\varphi_{m,n}\right)SIF\left(\alpha_{j,k},\varphi_{m,n},{\bar{\varphi }}_{n},M_{n},M_{n}^{\prime \prime }\right)\}$$

which can be recast in matrix form as

(20) $$\underline{\underline{K}}\underline{U}=\underline{NU}$$

where $\underline{NU}$ is the column vector of the NF samples reconstructed via the cylindrical wave correction on the nominal cylindrical surface and affected by known 2-D mispositioning errors, $\underline{U}$ is the column vector of the NR samples to be estimated, and $\underline{\underline{K}}$ is a sparse matrix, whose elements are given by the OSI kernel and has dimensions equal to R×R. This system can be conveniently resolved through the iterative scheme [37]. This last is derived by splitting the matrix $\underline{\underline{K}}$ in terms of its diagonal ${\underline{\underline{K}}}_{D}$ and non-diagonal $\underline{\underline{\Delta }}$ parts, by multiplying both terms of (20) by ${\underline{\underline{K}}}^{-1}$, and, finally, rearranging the terms, thus obtaining:

(21) $${\underline{U}}^{\left(\upsilon \right)}={\underline{\underline{K}}}_{D}^{-1}\underline{NU}-{\underline{\underline{K}}}_{D}^{-1}\underline{\underline{\Delta }}{\underline{U}}^{\left(\upsilon -1\right)}={\underline{U}}^{\left(0\right)}-{\underline{\underline{K}}}_{D}^{-1}\underline{\underline{\Delta }}{\underline{U}}^{\left(\upsilon -1\right)}$$

where ${\underline{U}}^{\left(\upsilon \right)}$ is the column vector of the NR samples evaluated at the υ-th iteration. The necessary condition [37] for the iterative procedure convergence can be considered satisfied since, according to the assumed hypotheses on the mispositioning errors distribution, it results ∀i $K_{ii}\neq 0$ and ∀r≠i $\left|K_{ii}\right|>\left|K_{ir}\right|$ and $\left|K_{ii}\right|>\left|K_{ri}\right|$. Finally, by making relation (21) explicit, it results:

$${\tilde{V}}^{\left(\nu \right)}\left(\sigma_{n},\varphi_{m,n}\right)=\frac{1}{SIF\left(\xi_{n},\sigma_{n},\bar{\sigma },N,N^{\prime \prime }\right)SIF\left(\alpha_{m,n},\varphi_{m,n},{\bar{\varphi }}_{n},M_{n},M_{n}^{\prime \prime }\right)}\cdot$$

(22) $$\begin{array}{l} \cdot \{\tilde{V}(\xi_{n},\phi_{m,n})-\sum_{k=k_{0}-q+1}^{k_{0}+q}\sum_{j=j_{0}-p+1}^{j_{0}+p}SIF\left(\xi_{n},\sigma_{k},\bar{\sigma },N,N^{\prime \prime }\right)SIF\left(\alpha_{m,n},\varphi_{j,k},{\bar{\varphi }}_{k},M_{k},M_{k}^{\prime \prime }\right){\tilde{V}}^{\left(\nu -1\right)}\left(\sigma_{k},\varphi_{j,k}\right)\}\;\\ \;\;\;\;\;(k\neq n)\wedge (j\neq m) \end{array}$$

where

(23) $$\begin{array}{lll} j_{0}=\{\begin{array}{cc} m & \mathrm{if}\phi_{m,n}\geq \varphi_{m,n}\\ m-1 & \mathrm{if}\phi_{m,n}<\varphi_{m,n} \end{array} & ; & k_{0}=\{\begin{array}{cc} n & \mathrm{if}\tau_{n,n}\geq \sigma_{n,n}\\ n-1 & \mathrm{if}\tau_{n,n}<\sigma_{n,n} \end{array} \end{array}$$

The reconstructed NR NF samples can be then interpolated through relation (11) to obtain the NF data required by the classical probe compensated cylindrical NTFF transformation [12].

## 4. Numerical Tests Results

Several numerical results assessing the effectiveness of the proposed two-step procedure to correct the 3-D positioning errors, which affect the antenna characterization in a cylindrical NF facility, are provided in this section. The numerical analysis refers to two different AUTs and to both of the considered models for elongated antennas.

The former antenna (AUT 1) is a uniform planar array whose elements are *z*-polarized Huygens sources, 0.7 *λ* and 0.8 *λ* spaced along *x* and *z*, respectively. Such an array covers the surface in the plane *y* = 0, delimited by an ellipse having semi-major and semi-minor axes equal to 20 *λ* and 6 *λ*. To provide effective modeling of this kind of source, a prolate spheroid with $a_{p}$ = 20 *λ* and $b_{p}$ = 6 *λ* has been adopted.

The latter (AUT 2) is again a uniform planar array of elementary Huygens sources, having the same polarization as the previous one, but now its elements, spaced by 0.5 *λ* along *x* and 0.7 *λ* along *z*, cover a surface in the plane *y* = 0 formed by a 12λ×30λ rectangle terminated by two half circles of radius 6 *λ*. Accordingly, this AUT has been modeled as enclosed in a rounded cylinder with $h_{rc}$ = 30 *λ* and $d_{rc}$ = 6 *λ*.

For both the AUTs, the NF samples have been generated as collected over a cylinder with a radius of 12 *λ* and a height of 140 *λ* by an open-ended WR-90 rectangular waveguide. The operating frequency is 10 GHz. Furthermore, to replicate actual measurements, the simulated samples consider the presence of 3-D probe mispositioning errors. More in detail, their radial coordinates present deviations from the radius *d* preset for the scanning, which are modeled as random variables uniformly distributed in the range [–0.1 *λ*, 0.1 *λ*]. Moreover, the non-uniform sampling points have been shifted in *φ* and *σ* regarding the related uniform sampling points. The deviations have been modeled as random variables with uniform distributions in the ranges $\left[-\Delta \varphi_{n}/3,\Delta \varphi_{n}/3\right]$ and [−Δσ/3,Δσ/3], respectively.

**Figure 4:**
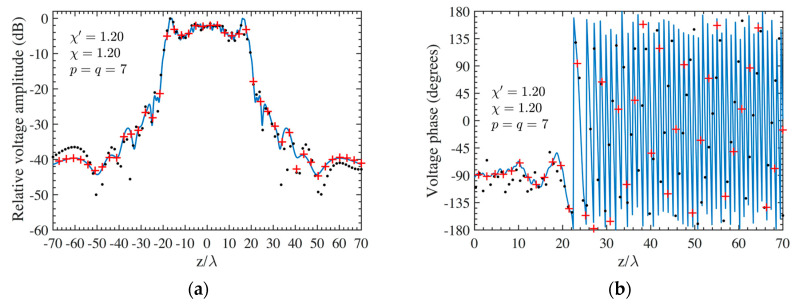
AUT 1. Voltage along the generatrix at *φ* = 90°. ––––– reference. ++++ achieved from the mispositioning errors affected NF samples by performing the devised strategy. •••• attained from the mispositioning errors affected NF samples without performing the devised strategy: (**a**) Amplitude; (**b**) Phase.

**Figure 5:**
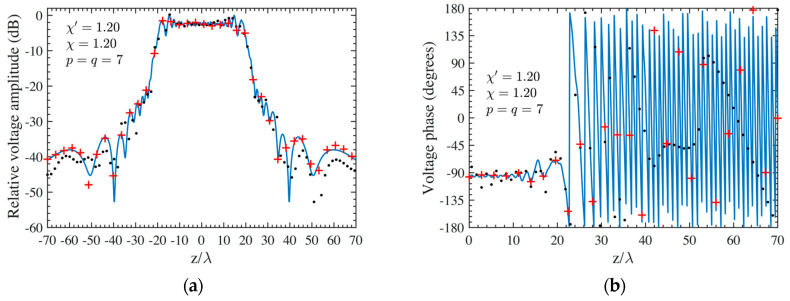
AUT 2. Voltage along the generatrix at *φ* = 90°. ––––– reference. ++++ achieved from the mispositioning errors affected NF samples by performing the devised strategy. •••• attained from the mispositioning errors affected NF samples without performing the devised strategy: (**a**) Amplitude; (**b**) Phase.

**Figure 6:**
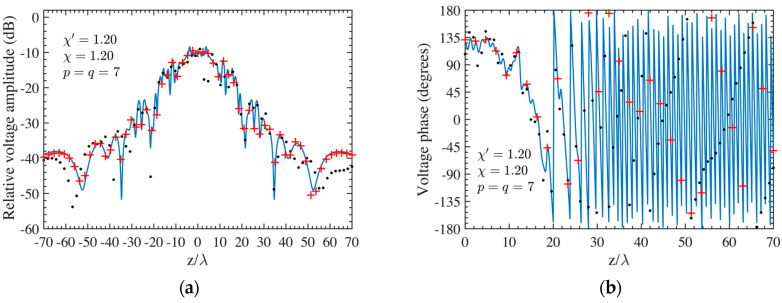
AUT 1. Voltage along the generatrix at *φ* = 60°. ––––– reference. ++++ achieved from the mispositioning errors altered NF samples by performing the devised strategy. •••• attained from the mispositioning errors altered NF samples without performing the devised strategy: (**a**) Amplitude; (**b**) Phase.

**Figure 7:**
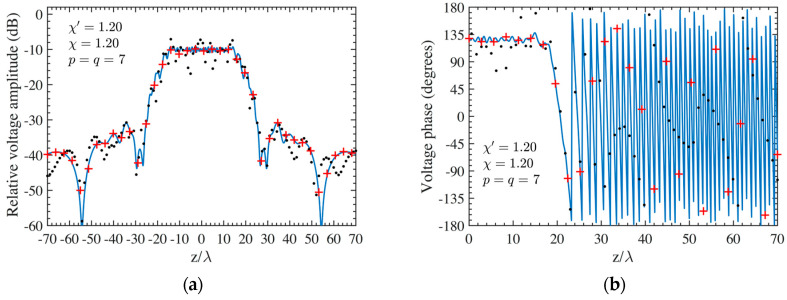
AUT 2. Voltage along the generatrix at *φ* = 60°. ––––– reference. ++++ achieved from the mispositioning errors altered NF samples by performing the devised strategy. •••• attained from the mispositioning errors altered NF samples without performing the devised strategy: (**a**) Amplitude; (**b**) Phase.

**Figure 8:**
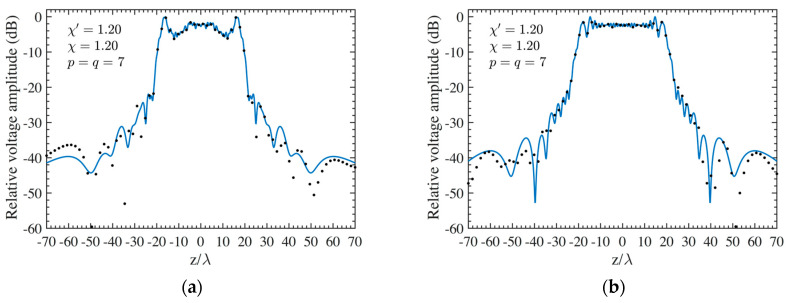
Voltage amplitude along the generatrix at *φ* = 90°. ––––– reference. •••• achieved from the mispositioning errors altered NF samples by performing only the phase correction: (**a**) AUT 1; (**b**) AUT 2.

**Figure 9:**
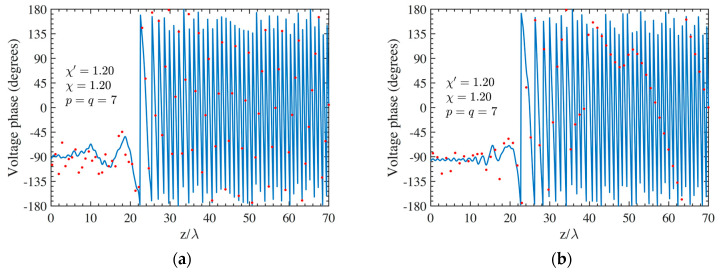
Voltage phase along the generatrix at *φ* = 90°. ––––– reference. •••• achieved from the mispositioning errors altered NF samples by performing only the iterative procedure: (**a**) AUT 1; (**b**) AUT 2.

As the first step, NF reconstructions are performed to assess the effectiveness of the developed procedure. Therefore, the amplitude and phase voltage patterns (solid line) along the generatrix at *φ* = 90° recovered from the mispositioning errors affected NF data, by using first the cylindrical wave correction in (18) and then the iterative technique, are shown in Figure 4a,b for the AUT 1, and in Figure 5a,b for the AUT 2, as compared with the exact ones and with those reconstructed by directly interpolating the NF samples affected by 3-D mispositioning errors. Furthermore, for the sake of completeness, the similar comparisons relevant to the cylinder generatrix at *φ* = 60° are reported in Figure 6a,b (AUT 1) and Figure 7a,b (AUT 2). Despite the severe condition enforced on the 3-D mispositioning errors, the developed strategy allows one to attain precise NF reconstructions. It should be pointed out that, to guarantee the convergence of the algorithm with very low errors [37], 10 iterations have been used in both cases for the solution of the linear system (20). To further highlight the necessity to use, besides the phase correction, the iterative-based approach, Figure 8a (AUT 1) and Figure 8b (AUT 2) show the amplitude of the voltage recovered along the generatrix at *φ* = 90° by employing only the cylindrical wave correction and not applying the iterative scheme. As can be seen, the reconstructions appear severely worsened. To conclude the NF analysis, the recovery of the phase of the voltage along the same generatrix obtained without performing the cylindrical wave correction before the use of the iterative-based approach is shown in Figure 9a and Figure 9b, for AUT 1 and AUT 2, respectively. It is evident that these reconstructions are seriously impaired too. The related amplitude reconstructions are not reported here to save space, but they appear to be less degraded. According to the above results, it should appear clear that the cylindrical wave correction and the iterative-based approach have to be necessarily matched in order to achieve effective compensation for the 3-D probe mispositioning errors.

**Figure 10:**
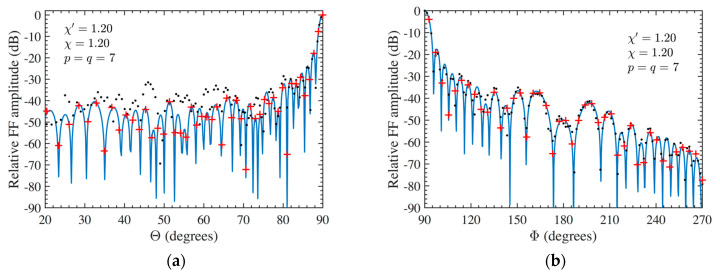
AUT 1. FF patterns in the principal planes. ––––– reference. ++++ achieved from the mispositioning errors altered NF samples by performing the devised strategy. •••• got from the mispositioning errors altered NF samples without performing the devised strategy (**a**) E-Plane; (**b**) H-plane.

**Figure 11:**
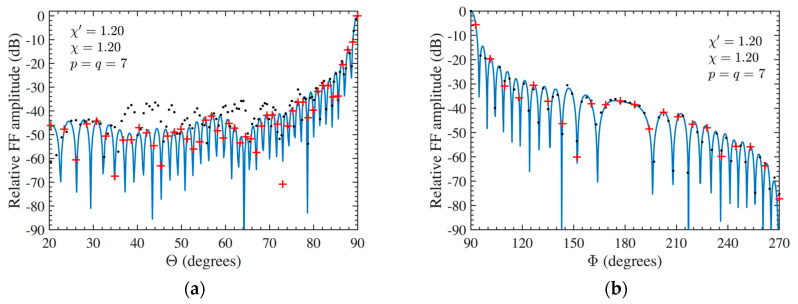
AUT 2. FF patterns in the principal planes. ––––– reference. ++++ achieved from the mispositioning errors altered NF samples by performing the devised strategy. •••• got from the mispositioning errors altered NF samples without performing the devised strategy (**a**) E-Plane; (**b**) H-plane.

**Figure 12:**
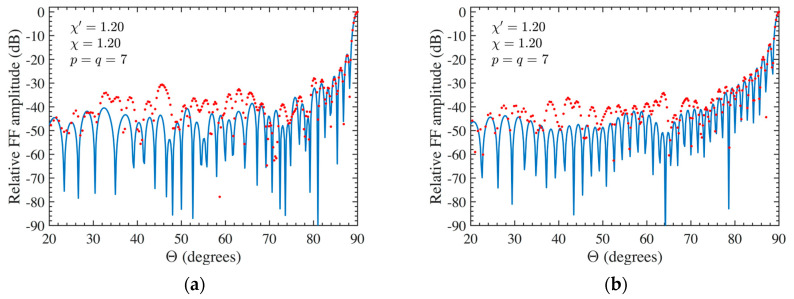
E-plane far-field pattern. ––––– reference. •••• got from the mispositioning errors altered NF samples by performing only the iterative technique: (**a**) AUT 1; (**b**) AUT 2.

At last, the FF reconstructions can be used to further demonstrate the effectiveness of the developed approach. To this purpose, the FF patterns in the principal planes E and H recovered by applying the developed two-step technique to the 3-D mispositioning errors altered NF data are compared in Figure 10a,b for AUT 1 and in Figure 11a,b for AUT 2, with the exact ones (references). Furthermore, the FF patterns, calculated from the mispositioning errors altered NF data when the compensation procedure is not applied, are shown in these figures. From them, it can be appreciated that, by applying the developed procedure, it is possible to attain very satisfactory FF reconstructions in both the principal planes in presence of significant 3-D mispositioning errors. On the other hand, the FF patterns recovered without applying the two-step technique appear severely deteriorated, in particular that in E-plane. Finally, the E-plane pattern recovery achieved by exploiting the 3-D mispositioning errors corrupted cylindrical NF data when only the iterative procedure is applied (without phase correction) is shown in Figure 12a and Figure 12b for AUT 1 and AUT 2, respectively. As can be observed, the recovery results are drastically worse in such a case. Therefore, the iterative technique alone does not allow us to successfully compensate for the 3-D probe positioning errors, and accordingly, it must be properly matched with the phase correction step.

To conclude, it is useful to compare the number of the needed NF samples, namely 11,117 for the AUT 1 and 14,006 for the AUT 2, to that (35,840) which would be required by the NTFF transformation using the classical cylindrical scanning.

## 5. Conclusions and Future Developments

An efficient two-step procedure to compensate for probe positioning errors, which may occur in the NR cylindrical NTFF transformation for elongated antennas based on both the prolate spheroid and rounded cylinder modeling of the AUT, has been proposed and numerically validated in this paper. The former step is a suitable phase correction that allows the compensation of the errors due to the deviations from the nominal scanning cylinder; the latter exploits an iterative technique to retrieve the correctly positioned samples from the non-uniform ones restored after the application of the former step. The reported numerical results have shown that, by applying the proposed two-step procedure, it is possible to obtain very accurate FF reconstructions even in the presence of severe 3-D positioning errors, whereas those attained when only the cylindrical wave correction or the iterative technique alone is applied result in significant deterioration. In the future, the proposed technique will be experimentally validated at the Antenna Characterization Lab of the University of Salerno in order to assess its practical feasibility.

## Data Availability

Data are contained within the article.
